# Pathogenicity and virulence of the blast fungus *Magnaporthe oryzae*

**DOI:** 10.1080/21505594.2026.2678667

**Published:** 2026-05-21

**Authors:** Camilla Molinari, Nicholas J. Talbot

**Affiliations:** The Sainsbury Laboratory, University of East Anglia, Norwich Research Park, Norwich, UK

**Keywords:** Appressorium, plant pathogen, rice blast, fungus, effector

## Abstract

The blast fungus *Magnaporthe oryzae* is the causal agent of the most serious disease of cultivated rice and an emerging threat to wheat production. Controlling blast diseases is therefore critical to ensuring global food security. In this review, we describe the mechanism by which the fungus ruptures the plant cuticle to gain entry to host cells and the virulence determinants necessary for suppression of host immunity and rapid colonization of plant tissue.

## Introduction

Fungal plant diseases account for 10–20% annual crop yield losses – enough food to feed four billion people each year [1]. This highlights fungi as a major threat not only for human health but also for modern agriculture, impacting a total of 168 crops identified as globally important by the Food and Agriculture Organisation (FAO) and thereby threatening global food security [2,3]. Controlling fungal diseases of crops is also challenging because of the rapid breakdown of genetically encoded disease resistance, often dependent on single dominant resistance genes, and the capacity of fungi to rapidly develop resistance to fungicides [4,5]. The blast fungus *Magnaporthe oryzae* (synonym of *Pyricularia oryzae*) is a filamentous ascomycete fungus [6–8], best known as the causal agent of rice blast disease, a disease responsible for destroying enough rice each year to feed more than 60 million people. The pathogen is, however, capable of infecting over 50 different cereals – including rice (*Oryza sativa*), barley (*Hordeum vulgare*), wheat (*Triticum aestivum*), and finger millet (*Eleusine coracana*) – across temperate and tropical regions, posing a significant threat to many staple crops that are particularly important in the developing world [9,10].

Among fungal pathogens, *M. oryzae* is especially devastating. This is because of its capacity to infect different host tissues, including leaves, nodes, stems, spikes, and panicles, causing distinct pathologies that can dramatically reduce the yield of crops, or even kill plants at the seedling stage. As a consequence, the blast fungus has been ranked among the most harmful fungal pathogens in the world [11]. Rice blast is most devastating in the Indo-Pacific Region, which contributes to 90% of world rice production [12], but responsible for destroying 10% to 30% of the annual rice harvest in more than 85 countries, including severe outbreaks in sub-Saharan Africa and South America, as well as Europe and Asia, making it the most important and pervasive disease of cultivated rice [10,13]. *M. oryzae* also poses an additional concern because of its capacity for host jumps, which has contributed to major disease outbreaks beyond rice [14]. Wheat blast disease, for example, emerged in Brazil in 1985 following a host jump from a wild grass-infecting strain of the fungus, growing on *Lolium* [15], and has since spread into neighboring South American countries. Since 2016, however, two distinct wheat blast epidemics have been reported in Zambia and Bangladesh – introduced by spread of a clonal population of the fungus from South America – which has led to catastrophic crop loss in both countries [16–19]. These profound economic impacts of blast disease, along with the genetic tractability and genomic resources of the fungus and its hosts [20–22], have made *M. oryzae* a model organism for studying fungal pathogenesis, plant immunity, and host-pathogen co-evolution [23]. Consequently, an emerging understanding of the molecular basis of blast disease has developed over the last three decades [6,10,24–26] and laid the foundation for current studies.

The complex relationship between plants and fungi dates back to around 600 million years ago, when fungi moved from marine to terrestrial environments through symbiotic associations with early land plants [27,28]. During this long coexistence, fungal pathogens emerged, and many have evolved specialized infection strategies to breach plant defenses and facilitate tissue colonization. Filamentous fungi and oomycetes are unique among plant pathogens (which include bacteria, nematodes, and viruses) in being able to penetrate the surface layers of plants and enter plant cells using infection structures – including appressoria, hyphopodia, penetration hyphae, haustoria, and rhizomorphs [29–34]. These structures are distinct from developmental stages observed in human-pathogenic fungi, which is likely a consequence of most fungal infections of humans arising opportunistically. They are often caused by fungi that have longstanding commensal associations with plants or which live by decaying plant material [35,36], such that they are not dependent on host infection for survival [37]. Another sophisticated strategy deployed by plant-pathogenic fungi is the secretion of large batteries of specialized proteins called effectors. These secreted effector proteins enable suppression of host immunity and facilitate fungal proliferation in host tissue [25,38–41]. The rapid evolution and diversification of effector-encoding genes is a characteristic of many plant pathogenic fungi, particularly those that grow in living plant tissues, such as biotrophs and hemibiotrophs. The effector repertoire of a plant pathogen appears to be directly related to its ability to infect its host [42]. *M. oryzae*, for example, is predicted to deploy over 500 different effector proteins during plant infection [43], which have evolved through gene duplication, diversification, genome rearrangements and even horizontal gene transfer [44–46]. This constant pathogen evolution also provides selective pressure for plants to adapt their immune systems to evade effector functions and thereby defend themselves from diseases, a dynamic which has been compared to a molecular “arms race” [47,48].

In this review, we describe key adaptations that the blast fungus has evolved to colonize its host, including morphogenetic changes required for initial plant infection and the deployment of effectors during growth *in planta*. We also compare what is known about *M. oryzae* in relation to fundamental virulence characteristics shared with other pathogens, including human pathogens. Our aim is to distinguish fundamental aspects of fungal pathogenesis, shared across numerous diverse pathogens, with specific adaptations to plant hosts and individual diseases, providing insight into the evolution of fungal pathogenesis.

## Early events of pathogenicity: Adhesion to the host and appressorium formation

Most fungal pathogens initiate disease by attachment to the host surface. Human-pathogenic fungi such as *Candida albicans* and *Aspergillus* species, for example, do so through the expression of adhesins – outer-surface components of the fungal cell wall [49,50]. Variability and expansion of adhesins encoded by pathogens suggest that these are context-specific and subject to tight regulation and leading to the idea that adhesins may follow a recognizable “code” that links sequence modules to adhesion phenotypes in a predictable manner [51]. Fungal adhesion is then often followed by secretion of depolymerizing enzymes that break down host barriers and morphological transitions, such as yeast-to-hypha transitions, that in some cases are used to breach host tissues [52]. By contrast, in *M. oryzae*, surface attachment to the host surface begins once a conidium – a three-celled asexual spore – lands on a rice leaf and attaches by secretion of an adhesive called spore tip mucilage [53]. This extracellular mucilaginous material is primarily composed of high-molecular-weight glycoprotein, similar to adhesives used by many fungi, as well as bacteria and algae, to facilitate surface associations [54,55]. Spore tip mucilage is, however, adapted to stick to the highly hydrophobic waxy cuticle, and the process also requires spermine produced by spermine synthases during conidial germination [56]. Adhesion is also enhanced through secretion of hydrophobin proteins, Mpg1 and Mhp1. Hydrophobins undergo interfacial self-assembly at hydrophobic surfaces or air-water interfaces, and in the context of appressorium development, they anchor attachment of the germ tube and incipient appressorium to the hydrophobic leaf surface by generating an amphipathic rodlet layer in which a hydrophilic surface is presented at the fungal cell wall for the action of adhesins and methyl esterases. This amphipathic boundary enables efficient appressorium development, underlining the importance of adhesion for infection-related morphogenesis [57–59]. Upon surface perception and hydration, the conidium germinates to form a narrow, polarized germ tube [60], which switches from anisotropic polar growth to form a flattened hook at the tip of the germ tube, differentiating to form an appressorium. This dome-shaped cell enables plant infection by physically breaching the host cuticle using a rigid penetration peg [10]. Formation of a functional appressorium is the most important prerequisite for establishing blast disease, pathogenesis by the blast fungus is therefore intrinsically related to host attachment, followed by a series of morphological changes that facilitate plant penetration [31,61].

Many fungi undergo morphological switches, changing from filamentous growth to propagation by yeast cells or vice versa, during their life cycle. Such dimorphic behavior enables some fungal species to reproduce, scavenge, shift from saprotrophic to pathogenic lifestyles, or establish symbiotic relationships [62]. These morphological transitions are typically prompted by environmental cues, perceived and translated internally through signal transduction cascades to result in modified growth. In the case of *Candida albicans*, for example, morphological changes have been extensively studied because its yeast-hypha transitions are key to its transition from a commensal to a systemic pathogen [63]. Virulence of *C. albicans* is therefore directly related to its ability to transition to hyphal growth, allowing host penetration, biofilm formation and the induction of host endocytosis [64–66]. By contrast, thermally dimorphic fungi, such as *Histoplasma* spp. and *Coccidioides* spp., switch from hyphal growth to a pathogenic yeast form driven by temperature [52]. These inducible morphogenetic switches are very similar to those found in filamentous fungi when a spore germinates into a hypha-like configuration, followed by penetration structure formation, which resembles a yeast-like growth stage, albeit one adapted to traverse a host surface. Such growth changes require switching from polarized growth to isotropic growth throughout various stages of infection [67]. Therefore changes in morphology are intrinsically linked to virulence in pathogenic fungi and, while signals triggering disease may differ among fungal pathogens – opportunistic human pathogens can, for example, become pathogenic when exposed to changes in temperature, CO_2_, and/or host-derived serum [68,69] – phytopathogens need to detect cues such as the plant cell wall, surface hydrophobicity, and/or plant hormones to induce morphogenetic changes [70,71]. In both cases, the underlying signal transduction cascades involved in differentiated fungal growth appear to be highly conserved, often involving Mitogen-activated protein kinase-mediated signaling [62].

## Signaling events required for infection-related development by *M. oryzae*

**Figure 1.**
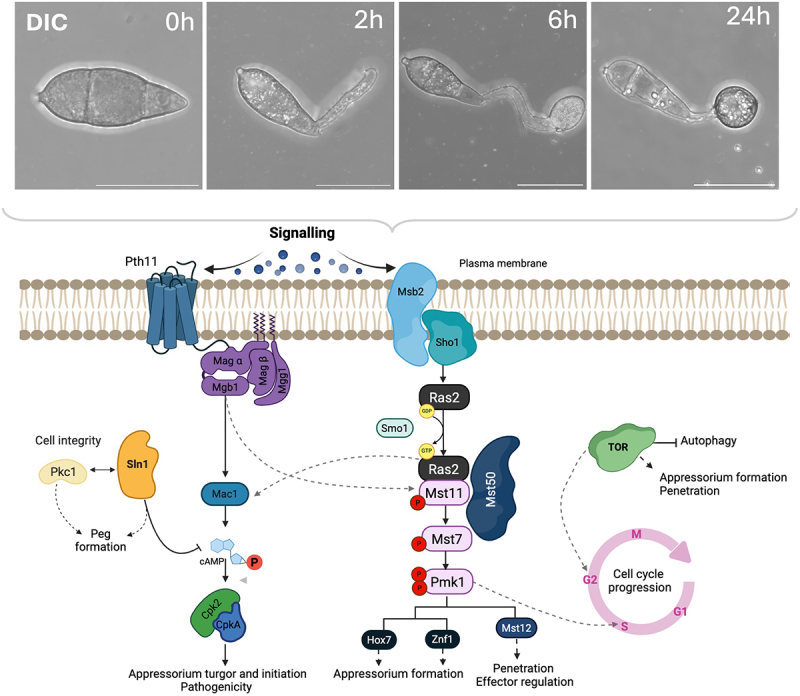
Signaling pathways associated with appressorium development in *M. oryzae*. The top panel shows a micrograph of *M. oryzae* appressorium development, captured at 0 hours (conidia before germination), 2 hours (elongation of the germ tube), 6 hours (incipient appressorium) and 24 hours (mature appressorium). The bottom schematic exemplifies the several interconnected signaling pathways needed for appressorium formation, penetration, and pathogenicity in the blast fungus. Pth11 acts as a sensor, triggering G protein dissociation and activating Mac1 adenylate cyclase, which produces cAMP to activate PKA and release CPKA for appressorium initiation and turgor. Sho1 and Msb2 sensors activate the Pmk1 MAPK pathway through Ras2, Smo1, and scaffold protein Mst50, leading to a kinase cascade (Mst11-Mst7-Pmk1) that regulates transcription factors (Hox7, Znf1, Mst12) essential for infection. Sln1 and protein kinase C1 (Pkc1) support penetration processes, while TOR signaling governs autophagy for recycling during infection. The formation and maturation of the appressorium are also tightly linked to cell-cycle progression.

*M*. *oryzae* utilizes two key signaling pathways to coordinate germination, appressorium formation and host penetration – the cyclic adenosine monophosphate (cAMP) response pathway, and the Pathogenicity MAP Kinase 1 (Pmk1) signaling pathway [6,72]. Appressorium development begins when the fungal germ tube detects a hard, hydrophobic surface and plant-derived chemical cues, such as waxes and cutin monomers. Since appressoria can form on hydrophobic plastic surfaces, the presence of plant signals is not essential to their development, but the presence of wax monomers is highly stimulatory to their formation. Surface perception occurs through G-protein coupled receptors (GPCRs) and sensor proteins [70,73]. The Pth11 receptor, for example, is a well-characterized GPCR known to promote appressorium formation and maturation [74], although its cognate ligand has not been identified. The activation of this sensor triggers dissociation of heterotrimeric G proteins (G_α_ and G_β_/G_γ_ sub-units), and activates the adenylate cyclase Mac1, which produces cAMP to activate protein kinase A (PKA) [75]. Release of the catalytic subunit CPKA from PKA is essential for appressorium maturation [76], as *ΔcpkA* mutants form small, nonfunctional appressoria. In parallel, other sensor proteins, including Sho1, a plasma membrane protein with four transmembrane helices and a cytosolic SH3 domain, and Msb2, a type I transmembrane mucin-like glycoprotein with a large, heavily glycosylated extracellular domain and single transmembrane domain, are required to activate the Pmk1 MAPK pathway via the GTP-binding protein Ras2 which interacts with the MAPKKK Mst11 and the adaptor protein Mst50, leading to the activation of the Pmk1 kinase cascade [33]. See Figure 1 for a schematic representation of the signaling pathway. This signaling complex, which includes the Mst11 MAPKKK, the Mst7 MAPKK and the Pmk1 MAPK, is homologous to the yeast MAPKKK Ste11-MAPKK Ste7-MAPK Fus3/Kss1 [77] involved in pheromone signaling, and in *M. oryzae* regulates a large number of transcription factors necessary for appressorium development. Hox7 and Znf1, for example, are required for appressorium initiation and formation [78,79], and Mst12 is essential for appressorium-dependent host penetration [80]. The central significance of the Pmk1 signaling pathway is supported by the fact that *Δpmk1* mutants are unable to cause blast disease and cannot make appressoria [72]. Furthermore, global transcriptional profiling has provided evidence that more than 6500 genes are ultimately controlled by the presence of Pmk1 during appressorium development [78,81]. A comprehensive phospho-proteomic analysis conducted during appressorium development of *M. oryzae*, identified 8,005 phosphosites on 2,062 fungal proteins, including a significant number of Pmk1-dependent phosphorylation events. Parallel reaction monitoring identify 32 Pmk1 substrates, including a novel regulator Vts1, which is essential for rice blast disease, in addition to confirming that Pmk1 directly phosphorylates transcriptional regulators, including Hox7, Znf1 and Mst12 [81]. Furthermore, a subset of the identified phosphosites is conserved across diverse fungi, with some present exclusively in plant-associated fungi and others more specifically in appressorium-forming species or those forming melanized infection cells. This provides evidence that Pmk1-dependent signaling may reflect an ancestral signaling pathway associated with invasive growth and pathogenesis, from which specific niche-specific differences have evolved for distinct lifestyles and pathologies [81]. Consistent with this, Pmk1 homologues have been functionally characterized in more than 30 fungal pathogens and shown to be important for disease, even when pathologies are very distinctive [82]. A known exception, however, is found for *Saccharomyces cerevisiae*, where filamentous invasive growth requires pheromone signaling regulated by MAPK KSS1 [83]. When considered together, these studies suggest that MAPK signaling is fundamental to the morphogenetic changes associated with plant infection, but is likely to be adapted from a much more conserved ancient function in regulating filamentous growth that is necessary for substrate invasion central to pathogenesis.

Appressorium formation and maturation are also tightly controlled by cell-cycle progression. During germ tube elongation, the apical cell of the conidium undergoes a single round of mitosis in which a newly formed daughter nucleus migrates to the tip of the germ tube, leading to appressorium differentiation and an asymmetric cytokinesis event that ultimately separates the infection cell from the germ tube [84,85]. The initiation of appressorium development requires an S-phase checkpoint via the DNA damage response pathway, followed by entry to G2 and mitosis, which are necessary for maturation of the appressorium. A second turgor-dependent S-phase checkpoint is then required for septin-associated appressorium repolarization [86]. These processes are coordinated with organelle trafficking to the appressorium, autophagy, and regulated conidial cell death [87,88].

## Appressorium maturation and host penetration

Appressorium-mediated plant infection by *M. oryzae* requires the generation of enormous turgor, which is directed as physical force to breach the plant cuticle [6,89]. Hydrostatic pressure results from the accumulation of glycerol and other polyols within the appressorium and the deposition of melanin in its cell wall. Melanin functions to enable water to diffuse into the appressorium but prevents larger solutes such as glycerol from escaping the infection cell, thus facilitating the buildup of osmotic pressure [33,90]. Fatty acid metabolism and the synthesis of glycerol, the critical osmolyte for turgor, are regulated through the cAMP – PKA signaling pathway [91]. Lipid body movement to the appressorium is meanwhile dependent on the Pmk1 MAPK pathway demonstrating again how coordination between the Pmk1 and cAMP-PKA pathways is necessary for virulence. Appressorium maturation also requires autophagy in the conidium and the recycling of the contents of the three-celled spore to the appressorium [92,93]. This also leads to nuclear degradation [94] of the three conidial nuclei, following mitosis. The Target of Rapamycin (TOR) signaling pathway has been implicated as a nutrient and stress sensor that regulates appressorium development and coordinates the control of autophagy and regulated cell death so that appressoria only form when conditions are appropriate. Consistent with this, rapamycin treatment affects appressorium development and pathogenesis [88]. It has been proposed that glutamine may act as an agonist of TOR and that a GATA transcription factor, Asd4, is required to limit intracellular glutamine levels. Loss of *ASD4* therefore increases glutamine, activates TOR, and thereby suppresses appressorium formation [88,95]. Autophagy is necessary for appressorium function because mutants affected in the process form nonfunctional appressoria that fail to re-polarize and cause infection. Regulated cell death of the conidium, which requires autophagy, may also require ferroptosis – an iron-dependent form of lipid peroxidation-driven cell death [96]. In *C. albicans*, both the RAS-PKA and TOR pathways are conserved and equally important for pathogenesis [97], highlighting that virulence in pathogenic fungi is coordinated based on nutritional sensing mechanisms that operate through similar molecular signaling pathways.

Repolarization of the appressorium and penetration peg formation requires major cytoskeletal rearrangements, as the cell changes its axis of polarity and develops a rigid penetration peg as the base of the appressorium perpendicular to the direction of germ tube growth [98,99]. This involves coordinated regulation of actin filaments, microtubules, and assembly of a septin ring, all of which contribute to host penetration [90]. Septins are GTPases that form higher-order structures based on heteropolymer formation. *M. oryzae* has four core septins, Sep3, Sep4, Sep5, and Sep6, which collectively form a septin ring at the appressorium pore, a defined region of the base of the cell from where the penetration peg emerges [100]. Cytoskeletal re-organization of the appressorium is regulated by the Pmk1 MAPK, the histidine-aspartate kinase Sln1, and the cell-wall integrity protein kinase C1 (Pkc1) [101–103]. The Sln1 kinase acts as a turgor sensor required for enabling the appressorium to switch from turgor generation to polarized growth and force generation. Mutants lacking Sln1 produce appressoria with excess turgor that fail to form a septin ring and repolarize [102]. Polarity factors, such as Cdc42, play a pivotal role in polarized growth and are deployed to the appressorium pore in a septin-dependent manner [99].

## Invasive growth and plant tissue colonization by *M. oryzae*

After cuticle penetration, primary invasive hyphae of *M. oryzae* rapidly fill the first invaded host cell, growing in a biotrophic manner [24,104]. Adjacent cells are then accessed through pit fields – aggregations of plasmodesmata, which are the cytoplasmic conduits between plant celld – undergoing severe hyphal constriction as they move across these cell-to-cell junctions. Movement between cells utilizes a specialized hyphal swelling called a transpessorium – an analogous structure to an appressorium – which also requires Pmk1 MAPK signaling [103,105]. *M. oryzae* has been classified as a hemibiotrophic pathogen, meaning that its ecological role lies along the spectrum between biotrophy and necrotrophy. The fungus initially establishes a biotrophic relationship with the host, where it occupies and feeds in living rice cells but does not kill them. In this context, it is surrounded by the invaginated host plasma membrane, which forms a compartment known as the extra-invasive hyphal membrane (EIHM) [106]. But later during leaf colonization, the fungus switches to necrotrophic infection, in which it kills host cells and uses their contents to fuel sporulation [10]. Hemibiotrophy in *M. oryzae* is critically linked to transporessorium activity. Initially colonized rice cells are always alive, for instance, but lose viability once a transporessorium infects an adjacent cell, because the fungal hypha ruptures the host plasma membrane upon entry to the next cell. By contrast to plant pathogens, human-infecting fungi such as *C. albicans*, *Aspergillus fumigatus*, *Cryptococcus neoformans*, *Histoplasma capsulatum*, and *Coccidioides immitis* are not known to have hemibiotrophic lifestyles. Instead, they primarily function as commensals, saprotrophs, or necrotrophs [107]. The only exception to this may be the pulmonary pathogen *Pneumocystis jirovecii*, a human fungal parasite that appears to act as an obligate biotroph, occupying living host cells, which has evolved to a very reduced genome that relies on host metabolism [108]. Within 96 hours of blast infection, small necrotic disease lesions appear on leaves and enlarge and coalesce during the next 2–3 days. At this point, conidiophores develop under conditions of high humidity, generating new conidia which are released, completing the asexual life cycle of *M. oryzae* [6].

## The rice blast fungus deploys a complex secreted proteome during plant infection

Fungi rely on an arsenal of secreted enzymes to acquire nutrients during pathogenesis. Depolymerizing enzymes, such as cellulases, hemicellulases, xylanases, cutinases, proteases, lipases, and oxidoreductases, break down complex polymers like cellulose, hemicellulose, lignin, cutin, proteins, and lipids in the external environment, enabling osmotrophic nutrient uptake and overcoming host barriers [109,110]. In phytopathogenic fungi such as *M. oryzae*, cell wall-degrading enzymes (CWDEs) are pivotal for breaching plant cell walls during infection, working in combination with appressorium turgor to facilitate entry and colonization [111]. Some degrading enzymes secreted by the blast fungus include CAZy (Carbohydrate-active enzymes), chitinases and pectinases, a family of glycosyl hydrolases (GH) that digest hemicellulose, and two endoxylanases (*MoXYL1A* and *MoXYL1B*) which digest xylan during infection [109,112,113]. Secretion of *MoXYL1A* and *MoXYL1B* has also been linked to maturation and functioning of the appressorium, in addition to later roles *in planta* [112]. Additionally, CWDEs such as polygalacturonases, endoxylanases, and cutinases not only degrade plant cell wall polymers but can also trigger defense responses or help fungi evade host immunity [109]. For example, oligosaccharides generated by different endoglucanases, such as the 3-β-D-Cellobiosyl-glucose and the 3-β-D-Cellotriosyl-glucose, are recognized by host immune receptors, acting as signaling molecules in plant-pathogen interactions [112]. In humans, pathogens like *C. albicans* also utilize batteries of secreted enzymes, such as aspartic proteases for nitrogen acquisition and to modulate host immunity [114], while *Cryptococcus neoformans* produces metalloproteinases to traverse host barriers in the brain [115]. The osmotrophic growth habit of fungal pathogens therefore implicates extracellular enzymes in virulence, to enable host barrier digestion and nutrient acquisition but also to inadvertently activate immunity signaling.

Fungi also secrete some biosynthetic enzymes to produce and/or elaborate secondary metabolites. Secondary metabolites have various ecological roles, acting as toxins, antibiotics, pigments, and modulating growth and/or providing environmental protection [116]. Typically, biosynthesis of secondary metabolites involves a primary synthase/synthetase that constructs the backbone of the metabolite, followed by additional enzymes that modify or decorate the initial structure. Primary enzymes include polyketide synthases (PKSs) that synthesize polyketides, non-ribosomal peptide synthetases (NRPSs) that assemble non-ribosomal, often cyclic peptides, terpene and cyclases (TSs and TCs) that synthesize terpenes, and diphosphate tryptophan synthases (DMATS) that produce alkaloids [117]. Plant pathogenic fungi encode a large number of these enzymes. *M. oryzae*, for instance, has a total of 22 PKS, 8 NRPS, 3 DMATS, and 10 PKS-NRPS hybrid enzymes – containing both a PKS enzyme and NRPS module [10,118]. With over 40 different genes involved in secondary metabolite biosynthesis – potentially including even more gene clusters yet to be discovered – the blast fungus possesses substantial metabolic capacity. In plant-infecting fungi, the abundance and diversity of these metabolic enzymes are reflected in their genomic adaptation, which is continuously driving virulence [52].

The abundance and variety of biosynthetic gene clusters encoding secondary metabolite synthesizing enzymes vary greatly between fungi and their ecological niche [119]. However, one shared metabolite that occurs across many fungal species, including *M. oryzae*, but also *Aspergillus fumigatus* and *Cryptococcus neoformans*, is melanin. There are two types of melanin, dihydroxynaphthalene (DHN) melanin and dihydroxyphenylalanine (DOPA) melanin, produced through different biosynthetic pathways [119]. DHN-melanin is necessary for appressorium function in *M. oryzae* [33] and for the human-infecting fungus *Aspergillus fumigatus*, where it protects the fungus from the action of human phagolysosomes and modulates host cytokine response [119,120]. DOPA-melanin, produced by *Cryptococcus neoformans*, can also promote virulence in different murine models [121].

Another crucial strategy for host invasion by plant pathogens involves secretion of specialized proteins known as effectors. Fungal effectors were initially characterized genetically through the gene-for-gene model, which describes how a pathogen avirulence (*Avr*) gene product interacts with a plant resistance (*R*) gene product, triggering host immunity and thereby halting disease progression [122,123]. However, since not all *Avr* gene products are recognized by plant *R* genes, the term “effector” was broadened to include secreted proteins that may or may not be detected by the host [39,124]. CWDEs and secondary metabolite biosynthetic enzymes are generally not classified as effectors because they lack specific host targets and typically cannot lead to effector-triggered immunity [125]. Nevertheless, secondary metabolites themselves can be pivotal for virulence and capable of manipulating the host. For example, during *M. oryzae* infection, the avirulence gene *ACE1* encodes a polyketide synthase, and while the product of this synthase remains unknown, production of its associated secondary metabolite by the rice blast fungus was shown to be essential for successful invasion [126]. However, rice cultivars containing the *R* gene *Pi-33* can recognize the *ACE1*-produced metabolite, which remains unknown, but highlights the possibility that host immunity can also be activated through metabolite recognition [127].

## Suppression of plant immunity by *M. oryzae*

Plants respond to host invasion through a two-layered immune system [125]. The first layer of defense consists of pattern-recognition receptors (PPRs), which perceive and respond to pathogen-associated molecular patterns (PAMPs) to activate PAMP-triggered immunity (PTI) [128]. Pathogens respond to PTI by secreting effector proteins with host targets associated with PTI or plant defense [39,124]. For instance, effectors can prevent PAMP production, release, or perception and can impair host immune signaling [129]. The *M. oryzae* effector Slp1 (Secreted LysM Protein 1), for example, is secreted into the apoplast – the space between the fungal cell wall and host plasma membrane or EIHM – and there inhibits chitin perception by sequestering pathogen-released chitin, preventing PAMP recognition by the host chitin elicitor receptor (CEBIP) [130]. Plants, however, can respond to pathogen effectors through a second layer of defense known as effector-triggered immunity (ETI), previously observed genetically as the gene-for-gene *Avr-R* interaction [125]. ETI involves specific recognition of effectors by immune receptors, which are often intracellular Nod-like receptors or NLR proteins [47]. NLR activation triggers immune signaling responses, including ROS (Reactive Oxygen Species) generation, MAPK signaling, phytohormone production, transcriptional reprogramming, calcium fluxes and the activation of localized programmed cell death or a hypersensitive response, that effectively stop disease progression [47,131]. Some pathogens, including *M. oryzae*, also secrete effectors that counteract ETI. For instance, Avr-Pita is a metalloprotease secreted by the blast fungus, which is internalized into the host cytoplasm and specifically targets the mitochondrial electron transport chain to manipulate ROS production [132]. This continued molecular battleground selects for rapid adaptation of both the pathogen and its host, driving an evolutionary arms race. Pathogens develop large effector repertoires to suppress immunity, and the plant evolves recognition to these effectors, prompting pathogens to respond by losing or mutating the recognized effector to then regain virulence [47].

This co-evolutionary dynamic is not as commonly seen in human-infecting fungi and their hosts [133]. In fact, there are few examples of true effectors characterized in human pathogenic fungi; most secreted proteins are enzymes, with few exceptions, such as *C. albicans* candidalysin, a peptide toxin derived from Ece1p protein required for damaging host tissues [134], or *Aspergillus fumigatus* Aspf1, a cytotoxic ribonuclease that impairs host protein biosynthesis [135]. This comparative lack of an effector repertoire may be related to their opportunistic pathogenic status, where the ability to infect human hosts is driven by thermotolerance coupled with a variety of strategies including morphological plasticity (such as yeast – hypha switching), adhesins, proteases, cell wall remodeling, the ability to grow in low‑nutrient or hostile environments, and to evade host immune systems [136,137]. Humans, like other vertebrates, have an adaptive immune system, which will tailor specific antibodies upon exposure to pathogen antigens. This individualized response is slower but does not require inheritance of pathogen recognition, unlike plant innate immunity [133]. Therefore, differentiated host immune responses may drive evolution of fungal pathogens distinctly, resulting in an expansion of effector proteins for plant pathogens such as *M. oryzae*, and acquired host tolerance strategies for vertebrate-infecting pathogens.

## The effector repertoire of *M. oryzae*

**Figure 2.**
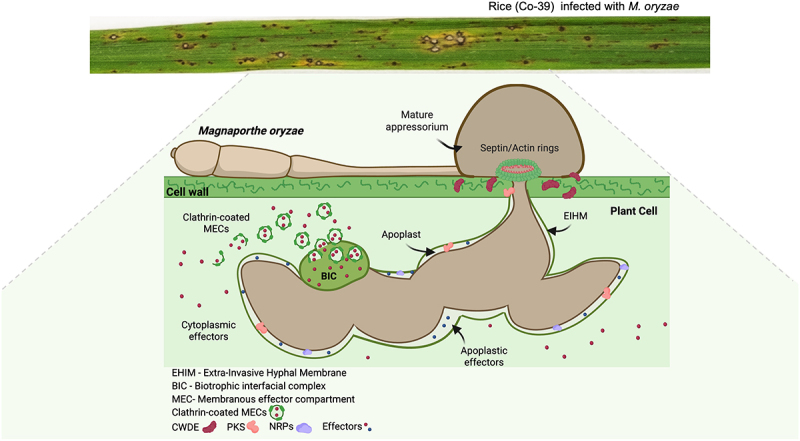
Invasive growth of *M. oryzae*. The top panel shows a rice leaf exhibiting typical blast lesions, rice (cultivar Co-39) infected with *M. oryzae* at 5 days post-inoculation. The bottom schematic illustrates the intracellular growth of *M. oryzae* within a host rice cell. A mature appressorium forms at the leaf surface, generating a penetration peg that breaches the plant cuticle and cell wall. Septin/actin rings organize at the appressorial pore to facilitate penetration. Fungal hyphae invade the host cell, surrounded by the extra-invasive hyphal membrane (EIHM) derived from the plant plasma membrane. The biotrophic interfacial complex (BIC) accumulates cytoplasmic effectors for delivery into the host cytoplasm, while apoplastic effectors are secreted into the surrounding apoplast. Additional cellular features depicted include plant clathrin involved in the internalization of cytoplasmic effectors, cell wall-degrading enzymes (CWDEs), polyketide synthases (pks), and non-ribosomal peptide synthetases (NRPs).

Effectors can be classified based on the cellular location to which they are delivered and their function. Apoplastic effectors act extracellularly in the apoplast – the space between the host cell wall and the plasma membrane – while cytoplasmic effectors are delivered into host cells, where they target specific signaling pathways and/or organelles [38–40]. In *M. oryzae*, apoplastic effectors are secreted through Golgi-dependent secretion, which is why some secreted enzymes have been considered apoplastic effectors. By contrast, delivery of cytoplasmic effectors occurs through a Golgi bypass pathway that requires exocyst function [138]. Cytoplasmic effectors accumulate in a plant membrane-derived structure known as the Biotrophic Interfacial Complex (BIC) before internalization [106,139,140]. Localization in the BIC appears to be diagnostic for cytoplasmic effectors and has been used in many studies to confirm the cellular destination of an effector [140,141]. It has also been shown that mutants lacking Uba4‑dependent tRNA thiolation, which are needed to decode AA‑ending codons efficiently, mis‑translate these codons, which disrupts cytoplasmic effector secretion into BICs and reduces virulence [142,143]. Cytoplasmic effector-encoding genes in *M. oryzae* therefore show over-representation of AA-ending codons, providing a potential bioinformatic route for their identification [144]. A recent study suggests that tRNA thiolation may even serve a wider role in the regulation of appressorium-specific gene expression [145]. How the BIC is utilized to facilitate effector uptake into rice cells is not entirely clear, but it has been shown that host clathrin-mediated endocytosis is required for effector uptake, suggesting that BICs are sites of effector internalization [146]. Further work is necessary to define the precise mechanism of effector delivery in *M. oryzae*, but the current model is shown in Figure 2.

The effector repertoire of a typical *M. oryzae* isolate, Guy11, has been shown to comprise 546 effectors, which corresponds to ~2% of the total proteome [43]. From this effector battery, ~30 effectors have been confirmed and/or functionally characterized [25]. The effector arsenal of *M. oryzae* includes proteins that help the fungus bypass plant immune recognition in the apoplast, such as Slp1 (Secreted LysM Protein 1) [130], and effectors that act within the host cytoplasm. Avr-Piz-t, for example, has multiple targets that interfere with both the plant proteasome and potassium uptake, ultimately suppressing PTI [147,148]. Other effectors function in transcriptional reprogramming. MoHTR1 and MoHTR2, for example, are delivered through the BIC and localize to the host nucleus, where they bind effector-responsive DNA elements in rice to alter gene expression [149]. Some effectors target other organelles: Avr-Pita, for instance, targets OsCOX11 in the mitochondrial electron transport chain [132]; MoPtep1 localizes to host peroxisomes [150]; while Avr-Pik and Pwl2 interact with host small Heavy Metal Associated (HMA)-domain proteins. The role of sHMAs in plant defense is not entirely clear, with some recent studies even suggesting a potential enzymatic function in cyclic nucleotide generation [151]. Though the role of Pwl2 appears to be to re-localize the HIPP43 sHMA from plasmodesmata into the cytoplasm, while Avr-Pik appears to stabilize its HMA targets HIPP19 and HIPP20 [141,152,153]. Interestingly, deleting sHMA-encoding genes reduces susceptibility to rice blast, while over-expression increases disease symptoms, suggesting that there may be susceptibility factors. Consistent with this, the product of *Pi21* is a sHMA, and *pi21* is a well-known recessive resistance gene for rice blast [154]. Other cytoplasmic effectors include BAS3, which localizes at cell wall junctions and may facilitate fungal spread between cells [155], and effectors that manipulate hormone pathways: MoIug4, for example, suppresses ethylene biosynthesis, while Iug6 and Iug9 affect both salicylic acid and ethylene signaling [156,157]. Additionally, MoNUDIX, an inositol pyrophosphate hydrolase, disrupts host phosphate sensing to promote plant growth and disease susceptibility [158], while the unconventional secreted protein GAS2 has been proposed to interact with rice kinase SnRK1, conferring enhanced susceptibility [159].

Despite these insights, most *M. oryzae* secreted proteins remain functionally uncharacterized, making high-throughput approaches to fully understand their roles in virulence vital for future studies [25]. This challenge is compounded by the rapid sequence diversification of effector genes, driven by the host – pathogen evolutionary arms race [47]. However, structural prediction tools have revealed convergence at the structural level even when sequences may differ, such as the case of MAX (*Magnaporthe* AVRs and ToxB-like) effectors [160], like Avr-Pik and Pwl2. MAX effectors often target sHMA domains and are co-regulated, revealing conservation in their molecular function during host colonization, which was not apparent from their gene sequences [25,43].

## Control strategies for blast disease

In spite of rapid progress in understanding the biology of plant infection by *M. oryzae*, control of blast diseases remains challenging and is only moderately effective. Existing management approaches integrate a combination of agronomic practices, chemical control, and genetic resistance [10]. However, with global temperatures continuing to rise, humidity-driven pathogens such as *M. oryzae* pose an escalating threat to global food security [161]. Environmental factors shape pathogen adaptation, reproduction, pathogenicity, and evolution. For *M. oryzae*, temperature is a key regulator of cell-cycle progression [162], drought reduces pathogenicity [163], and light and phosphate availability affect spore release and sexual reproduction [164,165]. Therefore, control strategies should also consider fungal adaptation to different abiotic stressors.

Agronomic practices which show partial efficacy in rice-growing regions include crop rotation, field sanitation, water management, and strain diversification [166]. Fungicide application has also played a significant role in rice blast management [167]. Commonly used chemical classes include triazoles, which inhibit the ergosterol biosynthesis pathway; strobilurins, which block the Qo site of complex III in the mitochondrial electron transport chain; and carboxamides, which interfere with fungal respiration and melanin biosynthesis [168]. The melanin biosynthesis inhibitor tricyclazole was also a very effective fungicide for *M. oryzae* [169,170], since it prevented appressorium function, but it has been withdrawn from many countries because the active ingredient has also been shown to have endocrine-disrupting activity [171,172]. However, resistance to these fungicides is increasingly reported due to point mutations, gene overexpression, and enhanced drug efflux regulation, each conferring varying degrees of tolerance [173–176]. Alternatively, plant and microbe-derived compounds, generally referred to as biocontrol strategies, might offer a safer environmentally compatible option to control blast disease [177]. Neem, an *Azadirachta indica* seed extract, for instance, can significantly inhibit in vitro fungal growth [178], while the use of beneficial bacteria, such as *Pseudomonas fluorescens*, can suppress blast disease by up to 60% in field trials [179]. However, biological off-target effects remain a major concern for the application of such approaches [180,181]. Other, less conventional approaches with protection potential include the use of externally applied RNAs (RNA interference) to inhibit essential fungal gene expression [182] or the application of external microbial elicitors, such as fungal chitin and chitosan, which can trigger activation of plant immunity, leading to systemic priming and protection [183]. However, production, stability, deployment, and trade-off effects of these pesticides remain major challenges [183,184]. Consequently, generating genetically resistant crop varieties remains the most promising approach for sustainable disease management [47,123,125].

To date, approximately 122 blast resistance (*R*) genes have been identified, of which around 39 have been cloned and functionally characterized [185], with considerable efforts to breed these into appropriate rice cultivars [186,187]. Among some of the characterized resistance gene interactions, the rice NLR (nucleotide binding leucine-rich repeat) protein pair Pi-CO39, formed of RGA4 and RGA5 proteins, confers resistance to Avr1-Co39 [188,189], the wheat genes Rmg7 and Rmg8 confer resistance to the same effector Avr-Rmg8 [190], the interaction of which has been mechanistically resolved at high resolution [191] shedding linght on how sensor-executor associatons occur and evolve, in a similar way to the the NLR pair Pik-1/Pik-2 which recognizes Avr-Pik effectors through a non-canonical integrated HMA domain in the NLR [192–194]. This has provided the foundation to engineer plant disease resistance through generating new NLR integrated domains such as those encoding custom-made nanobodies [195]. This provides a potential means to generate new resistance specificities and produce rice lines with durable blast resistance. However, despite these advances, rice blast disease currently persists due to the deployment of individual *R* genes that are often overcome by new pathogen races. Future strategies should therefore focus on gene pyramiding – the strategic combination of multiple resistance genes – coupled with pathogen pathotype monitoring to achieve broad-spectrum and durable resistance [154]. Such an approach has been trialed in sub-Saharan Africa, for example, with promising results that use marker-assisted selection to develop rice lines with multiple resistance specificities [180].

## Conclusion

The virulence mechanisms exhibited by the blast fungus *M. oryzae* have been selected for by its long association with grass hosts. Driving development of its pressure-dependent appressorium infection strategy as a consequence of needing to infect plants with tough waxy cuticles. This has clearly involved co-opting very conserved fungal signaling pathways that control invasive growth in very diverse species, including human pathogens. However, it has also evolved a repertoire of immune suppressive effectors to combat plant innate immunity mechanisms and is distinct in this regard from opportunistic human pathogenic species, but typical of plant pathogens, where such effector repertoires are common, particularly in biotrophs. Investigating virulence in the blast fungus has therefore provided new insight into the evolution of fungal pathogenesis in the context of a plant host [6,10,24–26].

Blast diseases remain a tough challenge for global agriculture, causing severe and recurrent losses in staple crops such as rice and wheat [9,10]. The capacity of *M. oryzae* to undergo host-jumps and rapid new host adaptations can lead to devastating epidemics in crops with few characterized sources of resistance [14]. The mechanism by which NLRs function in rice blast disease is, however, now understood in much greater detail than previously [196], with new strategies for NLR engineering that have considerable potential, including ingineering of NLRs to recognise multiple pathogenic effectors [197] or the integration of custom-made domains or nanobodies into the scaffolds of NLRs for recognition of specific effectors [195]. New approaches for NLR identification have also been reported, enabling rapid identification of new sources of resistance to wheat blast [198]. Analysis of the effector repertoire offers an alternative means to identify cognate R-genes that can be used in directed breeding programmes and genome editing approaches for more durable blast resistance. The challenge of blast does, however, underscore the need for continued research into the molecular basis of pathogenicity, plant immunity, and innovative disease management strategies.

## Data Availability

There is no data associated with this research
